# Emerging Mechanistic Links Between Fucosylation and Senescence in Lung Diseases

**DOI:** 10.70322/jrbtm.2026.10005

**Published:** 2026-06-23

**Authors:** Snehalakshmi Kavacheri Subramaniam, Minxuan Hu, Wenjing Gu, Angle Xu, Peisong Gao

**Affiliations:** 1 Division of Allergy and Clinical Immunology, Johns Hopkins University School of Medicine, Baltimore, MD 21224, USA; 2 Department of Respiratory Medicine, Children’s Hospital of Soochow University, Suzhou 215000, China

**Keywords:** Cellular senescence, Glycosylation, Fucosylation, Fucosyltransferases, SASP, asthma, COPD, IPF

## Abstract

Cellular senescence is increasingly recognized as a key driver of chronic lung diseases, contributing to persistent inflammation, impaired tissue repair, and pathological remodeling. In parallel, aberrant protein fucosylation has emerged as an important regulator of epithelial function and immune signaling in the respiratory tract. Recent evidence suggests that these processes may be mechanistically linked rather than independently dysregulated. In particular, core fucosylation mediated by fucosyltransferases, such as fucosyltransferases 8 (FUT8), can modulate receptor activity and amplify signaling pathways, including TGF-β/Smad and PI3K/Akt, that are central to the induction and maintenance of cellular senescence. These interactions may promote epithelial dysfunction, the senescence-associated secretory phenotype (SASP) production, and pro-fibrotic remodeling in diseases such as COPD, asthma, and idiopathic pulmonary fibrosis. In this review, we synthesize current knowledge on cellular senescence and fucosylation in chronic lung disease and highlight emerging evidence linking these processes through shared signaling networks. We further discuss the potential of the fucosylation-senescence axis as a source of novel biomarkers and therapeutic targets. This review is among the first to integrate emerging evidence linking aberrant fucosylation with cellular senescence signaling in chronic lung diseases, thereby providing a conceptual framework for future mechanistic and translational studies.

## 1. Introduction

Cellular senescence has emerged as an important contributor to the pathogenesis of chronic lung diseases, including chronic obstructive pulmonary disease (COPD) and idiopathic pulmonary fibrosis (IPF), largely through its roles in inflammation and tissue remodeling [1]. Senescence is a stable state of cellcycle arrest accompanied by extensive phenotypic reprogramming. In response to cumulative cellular stresses such as telomere attrition, oxidative stress, DNA damage, and mitochondrial dysfunction, proliferative cells activate canonical pathways, including the p53–p21 and p16^INK4A^-RB axes, which enforce durable growth arrest. Although senescent cells no longer divide, they remain viable and metabolically active, and secrete a diverse array of inflammatory cytokines, chemokines, and matrixremodeling factors collectively known as the senescence-associated secretory phenotype (SASP) [2–6]. These SASP factors drive persistent inflammation at both local and systemic levels [7–14]. In the lung, chronic environmental exposures, repeated epithelial injury, and impaired tissue repair promote the accumulation of senescent cells in epithelial, stromal, and other structural compartments. Sustained SASP signaling contributes to chronic inflammation, aberrant tissue remodeling, and fibrosis, thereby promoting the development and progression of COPD [15,16], asthma [17–20], allergic rhinitis [21], IPF [22], and pulmonary hypertension [23]. Although therapeutic targeting of senescence has shown promise in other chronic diseases [19,21,24,25], senescence is highly heterogeneous and context dependent, varying according to cell type, disease stage, and microenvironment [1,26,27]. As a result, the regulatory mechanisms that determine senescence phenotypes, particularly those controlling SASP composition and persistence, remain incompletely understood.

Glycosylation, the enzymatic process of adding carbohydrate moieties to proteins and lipids, is a highly dynamic and tightly regulated post-translational modification that profoundly influences epithelial biology [28–30]. In barrier tissues such as the lung, skin, and gut, epithelial glycosylation modulates a wide range of functions, including cell-cell adhesion [31], pathogen recognition [32], mucosal barrier integrity [33,34], and immune signaling [28,35,36]. Aberrant glycosylation patterns have been increasingly recognized as key contributors to disease pathogenesis [28,37]. In chronic inflammatory diseases such as asthma, inflammatory bowel disease, and cystic fibrosis, altered glycosylation of mucins and membrane-bound glycoproteins can disrupt epithelial barrier function, enhance susceptibility to microbial colonization, and drive persistent immune activation [29,32,34,36,38]. Despite its fundamental role in diverse diseases, the mechanisms regulating glycosylation in epithelial cells and its functional role in lung disease remain incompletely understood.

Fucosylation is a major form of glycosylation in which L-fucose is transferred from GDP-fucose to glycoproteins, glycolipids, or specific protein motifs by fucosyltransferases [39]. It plays a fundamental role in regulating biological processes such as cell signaling, adhesion, immune surveillance, and host– microbe interactions [29,34,40–44]. Fucose is present in approximately 20–90% of N-linked glycans on cell surface glycoproteins, highlighting its widespread involvement in epithelial biology [45]. Fucosylation is mediated by a family of fucosyltransferases (FUTs), comprising 13 human isoforms that differ in their substrate specificity and glycosidic linkages with distinct linkage specificities, including α1,2-, α1,3-, α1,4-, and α1,6-fucosylation [30,46–49]. Dysregulated fucosylation driven by different FUTs has been increasingly recognized in various disease contexts, including cancer, chronic inflammatory disorders, epithelial barrier disruption, epithelial-mesenchymal transition, and ECM-dependent tissue remodeling [50,51], and allergic diseases such as asthma [30,41,46–49,51–57].

An emerging but still underexplored question is whether aberrant fucosylation actively regulates senescence programs rather than merely reflecting chronic tissue injury. Given that cellular senescence is highly responsive to extracellular cues, matrix context, and stress-response signaling, glycan-dependent modulation of receptor function and cell-matrix interactions may directly influence the initiation, maintenance, and phenotypic output of senescence [58,59]. Supporting this concept, recent studies in pulmonary fibrosis have linked altered core fucosylation to pathways governing alveolar epithelial cell senescence. In particular, FUT8-mediated core fucosylation has been shown to enhance TGF-β signaling and promote epithelial cell senescence, whereas inhibition of FUT8 attenuates these effects [43]. Core fucosylation has also been implicated in the activation of IGF-1 and PI3K/Akt signaling pathways in IPF-associated epithelial senescence [60]. Collectively, these findings suggest that dysregulated fucosylation may reinforce senescence-associated signaling by modulating key receptor pathways, thereby sustaining epithelial dysfunction and driving pro-fibrotic remodeling. In this review, we discuss the roles of cellular senescence and fucosylation in chronic lung disease, examine the mechanistic intersections between these processes, and highlight emerging opportunities for biomarker development and therapeutic intervention. Importantly, this review provides a conceptual framework for future mechanistic and translational studies.

## 2. Cellular Senescence in IPF, Asthma, and COPD

In chronic lung diseases, senescence is increasingly recognized as a functionally important driver of disease progression rather than a mere bystander phenomenon. Senescent epithelial, mesenchymal, and vascular cells accumulate across diseased lung tissues and contribute to persistent inflammation, defective repair, and pathological remodeling [61–63]. These cells typically exhibit increased expression of cell-cycle inhibitors such as p16^INK4A^ and p21^CIP1^, elevated SA-β-gal activity, and enhanced production of SASP factors [64]. Much of their pathogenic effect is attributed to sustained SASP signaling, which amplifies local inflammatory responses, disrupts tissue homeostasis, and perpetuates chronic tissue injury. Senescence in chronic lung diseases is sustained by persistent activation of stress- and injury-responsive signaling pathways that couple environmental injury to aberrant repair and inflammatory remodeling, including TGF-β/Smad, Wnt/β-catenin, PI3K/AKT/mTOR, p38 MAPK, JAK/STAT, and NF-κB (Table 1). These findings indicate that senescence is mechanistically integrated with inflammation, epithelial dysfunction, and tissue remodeling across diverse chronic lung disorders.

## 3. Idiopathic Pulmonary Fibrosis: Senescence as a Core Profibrotic Driver

In IPF, cellular senescence is a central driver of fibrotic remodeling, involving both epithelial and mesenchymal compartments. Bronchial and alveolar epithelial cells, particularly alveolar type II (AT2) cells, exhibit senescence induced by TGF-β/Smad signaling, leading to impaired epithelial regeneration and sustained profibrotic signaling [65–68]. Notably, p53 activation in senescent AT2 cells establishes an autocrine TGF-β feedback loop, perpetuating fibroblast activation and myofibroblast differentiation [66]. Additional pathways amplify this senescence-fibrosis axis. Wnt/β-catenin signaling drives epithelial senescence and fibroblast activation via β-catenin-dependent transcriptional programs [73], while PI3K/AKT signaling, activated through PTEN loss or IGF1-mediated mechanisms [15,75], further promotes epithelial senescence and fibrotic progression. Concurrently, p38 MAPK activation following DNA damage induces SASP development [84], and JAK/STAT signaling contributes to both epithelial and fibroblast senescence [87,88]. NF-κB activation further reinforces inflammatory and senescence-associated responses [91]. Collectively, these findings position senescence as a key initiator and perpetuator of fibrosis in IPF.

## 4. Asthma: Senescence as a Mediator of Airway Inflammation and Remodeling

In asthma, the role of senescence is increasingly recognized in airway remodeling and epithelial dysfunction [1,18,92]. Unlike IPF, where fibrosis dominates, senescence in asthma primarily contributes to dysregulated airway inflammation and chronic airway structural changes. Several signaling pathways have been linked to the role of senescence in asthma (Table 1). Especially, deranged Wnt/β-catenin signaling in asthma is associated with abnormal senescence-related stress responses and dysregulated repair programs in bronchial epithelium and fibroblasts [74]. In airway epithelial cells, mTOR signaling mediates cigarette smoke-induced senescence and promotes asthma development [76]. In addition, TSLP-STAT3 signaling induces bronchial epithelial cell senescence and contributes to airway remodeling [90]. These observations place senescence at the intersection of epithelial injury, aberrant repair, and chronic type 2-skewed airway remodeling.

**Figure 1.**
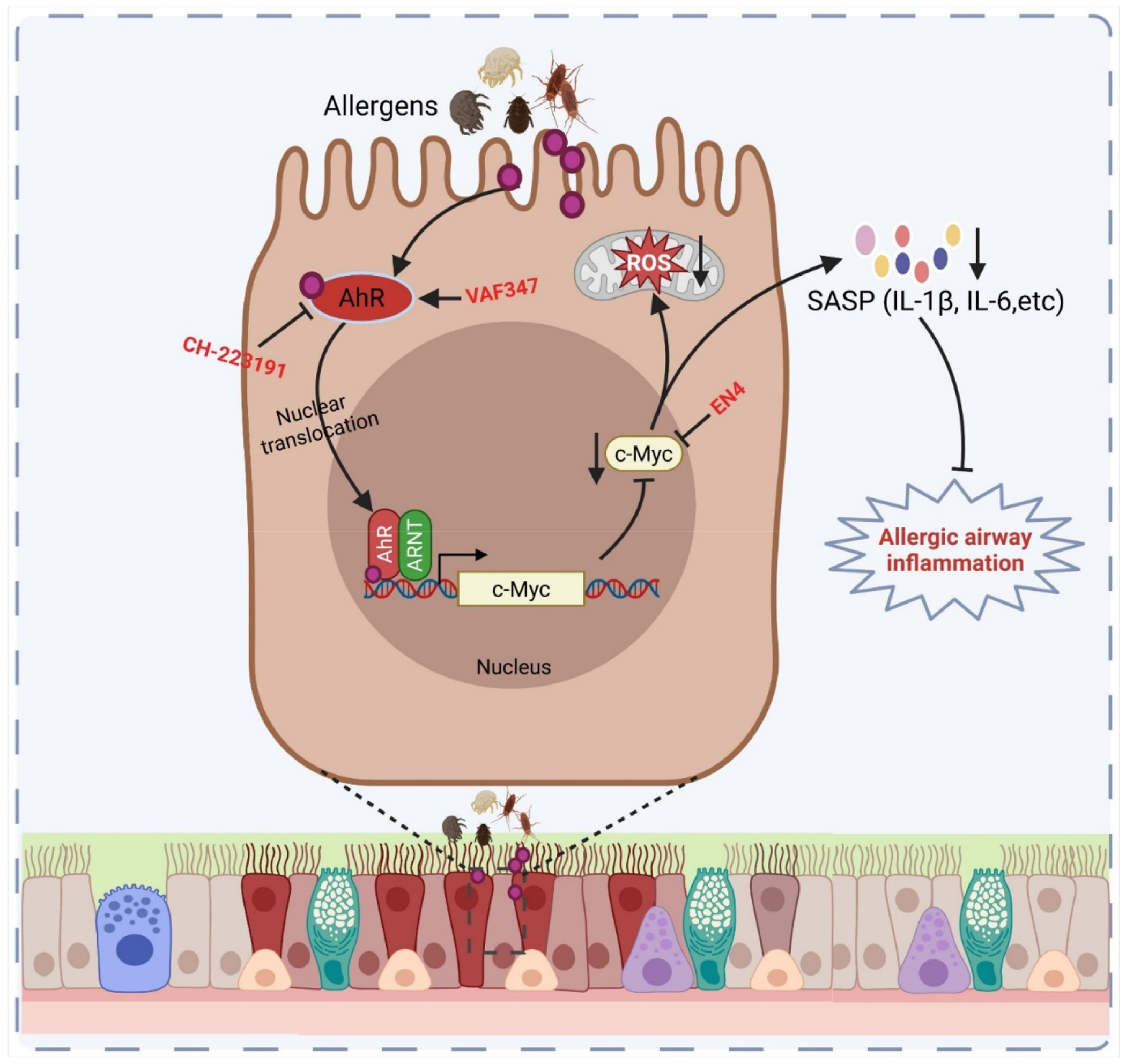
AhR-c-MYC-ROS signaling axis in allergen-induced airway epithelial senescence and inflammation. Environmental allergens activate AhR signaling in airway epithelial cells, promoting AhR nuclear translocation and transcriptional activation of downstream targets, including c-MYC. Enhanced c-MYC activity drives metabolic reprogramming and mitochondrial ROS production, leading to cellular stress and induction of senescence-associated secretory phenotype (SASP) factors such as IL-1β and IL-6. These proinflammatory mediators contribute to allergic airway inflammation and epithelial remodeling. Pharmacological inhibitors targeting AhR (e.g., CH-223191, VAF347) or c-MYC (EN4) attenuate pathway activation and downstream inflammatory responses [19].

A recent study identifies airway epithelial cellular senescence as a central pathological feature of allergen-induced asthma and defines a novel regulatory mechanism linking environmental sensing to senescence and inflammation via the AhR-c-Myc axis [19]. Mechanistically, the aryl hydrocarbon receptor (AhR) was identified as a critical protective regulator of epithelial homeostasis. Although AhR activity is increased following allergen exposure, likely as a compensatory response, its function is insufficient to counterbalance strong pro-senescent signals. Genetic deletion of AhR in airway epithelial (Club) cells exacerbates ROS generation, senescence, and airway inflammation, whereas pharmacologic activation of AhR suppresses these processes. Transcriptomic and mechanistic analyses further identify c-Myc as a key downstream effector of AhR signaling. Further study demonstrates that AhR directly binds to the c-Myc promoter and negatively regulates its expression. Allergen exposure induces c-Myc, which in turn promotes ROS production, epithelial senescence, and SASP-driven inflammation. Importantly, pharmacologic inhibition of c-Myc (EN4) attenuates epithelial senescence, reduces pro-inflammatory cytokine release (e.g., IL-1β, IL-6), and significantly improves airway inflammation *in vivo* (Figure 1). Overall, this study defines the AhR-c-Myc axis as a novel pathway controlling epithelial senescence and asthma pathology, highlighting senescence as a promising therapeutic target.

**Figure 2.**
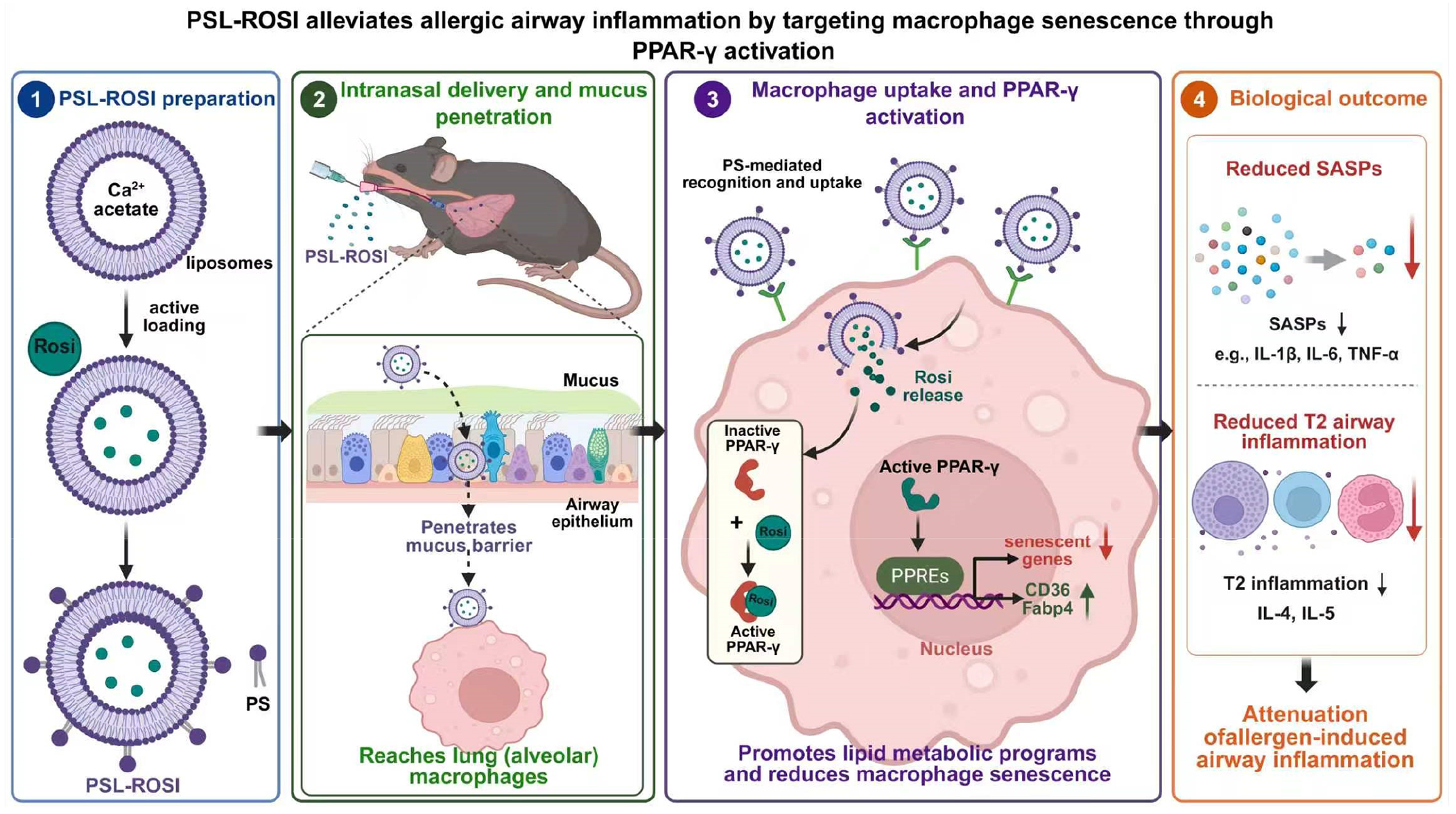
Macrophage-targeted PSL-Rosi nanoparticles deliver rosiglitazone via phosphatidylserine-mediated uptake, activating PPARγ signaling to suppress SASP production and attenuate type 2 airway inflammation [20].

Recently, macrophage senescence was recognized as a key driver of allergic airway inflammation in asthma [20]. Allergen exposure (cockroach extract) induces robust senescence in lung mononuclear phagocytes, particularly macrophages, as shown by increased SenMayo scores, p16 expression, and SA-β-Gal staining. Functionally, clearance of senescent cells (D&Q senolytics) significantly reduces airway inflammation, eosinophilia, mucus production, and Th2 cytokines (IL-4, IL-5), demonstrating a causal role for senescence in disease pathogenesis. Mechanistically, the study identifies peroxisome proliferator-activated receptor gamma (PPAR-γ) as a critical negative regulator of macrophage senescence. PPAR-γ expression is inversely correlated with senescence, and macrophage-specific PPAR-γ deletion exacerbates senescence and airway inflammation. Therapeutically, activation of PPAR-γ with rosiglitazone suppresses macrophage senescence, reduces SASP factors, and attenuates airway inflammation, both in vitro and in vivo. Targeted delivery using phosphatidylserine (PS)-modified liposomes loaded with rosiglitazone (PSL-Rosi) further enhances efficacy by delivering the drug directly to lung macrophages. Finally, integrative analyses reveal that PPAR-γ regulates macrophage lipid metabolism (e.g., CD36, FABP4), linking metabolic reprogramming to control of senescence and inflammation (Figure 2). Overall, the study establishes a novel PPAR-γ-lipid metabolism-senescence–inflammation axis (PPAR-γ-CD36/FABP4) in macrophages and highlights macrophage senescence as a promising therapeutic target in asthma.

Cellular senescence has also emerged as a critical mechanism linking epithelial stress to chronic airway inflammation and remodeling in allergic rhinitis [21]. In this study, epithelial dysfunction in AR is characterized by increased oxidative stress, Th2 inflammation, and structural remodeling, all of which are strongly associated with activation of the RhoA/ROCK signaling pathway. Elevated RhoA activity correlates with disease severity and drives epithelial cellular senescence, as evidenced by increased expression of canonical markers (*p16, p21, γH2AX*) and SASP factors, including IL-1β and IL-6. Mechanistically, RhoA-induced oxidative stress promotes mitochondrial dysfunction and suppresses PRKN (also known as PARK2/Parkin), a key regulator of mitophagy and mitochondrial homeostasis [93], thereby amplifying senescence and inflammatory signaling. Functional studies demonstrate that inhibition of RhoA/ROCK or genetic clearance of senescent cells significantly attenuates epithelial remodeling, oxidative injury, and Th2 inflammation, establishing senescence as an active driver rather than a bystander in disease pathogenesis. Importantly, restoration of PRKN reverses mitochondrial damage, reduces ROS accumulation, and suppresses epithelial senescence. Collectively, these findings define a novel RhoA-PRKN-senescence axis that integrates oxidative stress with epithelial remodeling and inflammation, highlighting both RhoA/ROCK signaling and mitochondrial quality control pathways as promising therapeutic targets in allergic airway disease. Collectively, senescence in airway diseases like asthma is especially important in airway inflammation and remodeling characterized by persistent epithelial damage, mucus metaplasia, subepithelial fibrosis, and steroid-refractory remodeling. Senescence is less a purely fibrotic endpoint than a remodeling-associated epithelial state that disrupts normal repair and perpetuates cytokine-driven pathology. Thus, senescence may help explain why some asthmatic airways fail to restore normal epithelial integrity and instead progress toward persistent airway inflammation and fixed structural changes.

## 5. COPD: Senescence as a Stress-Induced Mechanism of Airway Destruction

In COPD and emphysema, senescence plays a broad role in epithelial cells, small airway fibroblasts, and vascular-associated cells. Small airway fibroblasts from COPD patients exhibit senescence together with increased TGF-β and Wnt/β-catenin signaling, suggesting impaired matrix homeostasis and abnormal repair [69]. Cigarette smoke-related pathways are prominent: FOXO1 promotes fibroblast senescence and fibrosis via TGF-β1/Smad2/3 signaling [70,71], while PDK1 inhibition suppresses PI3K/AKT-dependent epithelial senescence in emphysema [77]. Erythromycin also attenuates oxidative stress-induced senescence through PI3K/AKT/mTOR signaling, supporting the therapeutic relevance of this axis [78]. Senescence in COPD is not limited to airway structural cells. mTOR activation drives senescence in pulmonary endothelial and alveolar epithelial cells, contributing to emphysema, pulmonary hypertension, and inflammation [76]. Interferon-JAK/STAT signaling promotes bronchial epithelial senescence and chronic airway inflammation [89]. Interestingly, the p16 senescence pathway can promote AT2 proliferation and regeneration through IGF1/Akt signaling in emphysema [79], highlighting that senescence-associated pathways may have context-dependent effects, including compensatory or maladaptive regeneration. Overall, COPD is a disease in which chronic smoke and oxidative stress induce a senescence program that undermines epithelial maintenance, disrupts matrix balance, and promotes both parenchymal destruction and chronic inflammation. Taken together, cellular senescence should be viewed as a central pathogenic mechanism in chronic lung disease rather than a secondary epiphenomenon. It integrates chronic injury with inflammatory signaling, impaired regeneration, and tissue remodeling. IPF shows the clearest senescence-fibrosis axis, asthma highlights senescence-driven airway remodeling and abnormal epithelial repair, and COPD emphasizes stress-induced senescence linked to inflammation and tissue destruction. Despite these disease-specific patterns, the repeated involvement of TGF-β/Smad, Wnt/β-catenin, PI3K/AKT/mTOR, p38 MAPK, JAK/STAT, and NF-κB suggests that senescence may represent a common therapeutic node across chronic lung diseases.

## 6. Fucosylation and Key Lung Disease Pathogenesis

Fucosylation has emerged as a critical regulator of cellular signaling and tissue homeostasis in chronic lung diseases [39,51]. By modifying glycoproteins, glycolipids, and receptors, fucosylation dynamically influences key biological processes, including epithelial barrier integrity, immune activation, and tissue remodeling [29,34,40–44]. Under physiological conditions, fucosylation supports epithelial barrier maintenance, coordinated repair responses after injury, mucin glycosylation, and epithelial-immune interactions [54,94]. For example, FUT2-dependent fucosylation strengthens E-cadherin-mediated intercellular adhesion, thereby preserving epithelial monolayer integrity and highlighting a direct role for fucosylation in airway epithelial homeostasis [95]. The expression of individual FUTs varies across tissues and cell types, reflecting their context-specific biological functions [39]. When dysregulated, however, these homeostatic programs can shift toward disease-promoting inflammation, defective repair, and tissue remodeling. Indeed, increasing evidence suggests that aberrant fucosylation is not merely a consequence of disease but actively contributes to the pathogenesis of conditions such as asthma, COPD, IPF, and pulmonary hypertension.

In asthma, increased epithelial fucosylation and upregulation of FUTs, including FUT2 and FUT8, have been linked to allergic airway inflammation, airway hyperresponsiveness, and mucus obstruction [52,55]. Enhanced α(1,2)-fucosylation promotes mucin glycosylation and can amplify inflammatory signaling through pathways such as complement activation (e.g., C3a) and dendritic cell activation, thereby sustaining Th2-driven inflammation [52]. This positions FUT2 as an upstream regulator linking epithelial barrier alterations to immune dysregulation in allergic airway disease. Golgi-localized FUT8 catalyzes N-glycan core fucosylatio of the glucose transporter GLUT1, which enhances epithelial glycolysis, thereby promoting airway inflammation and epithelial-mesenchymal transition in bronchial epithelial cells [45,57]. Current evidence of FUT1 in asthma is limited. Recent data suggest that it may act as a critical epithelial regulator in asthma, linking glycosylation remodeling to airway barrier dysfunction and allergic inflammation [96,97]. Evidence demonstrates that FUT1 expression and α1,2-fucosylation are markedly upregulated in the airway epithelium following allergen exposure, representing a coordinated epithelial stress response. Functionally, increased FUT1 activity disrupts epithelial junctional integrity by altering key adhesion molecules, such as ZO-1 and E-cadherin, thereby enhancing epithelial permeability and facilitating allergen penetration. This barrier breakdown is accompanied by increased release of epithelial-derived alarmins, including TSLP and IL-25, which amplify downstream Th2 immune responses. Collectively, this study identifies increased fucosylation, particularly α1,2-fucosylation, as a potential contributor to asthma pathogenesis. The elevated expression of Fut1 in both lung tissues and airway epithelial cells suggests a pivotal role for this enzyme in the abnormal glycosylation observed in asthma, warranting further investigation.

In COPD, chronic exposure to oxidative stress and cigarette smoke is associated with glycosylation changes, including dysregulated fucosylation, which may impair epithelial repair, alter mucus properties, and contribute to persistent inflammation and airway remodeling. Reduced FUT2- and FUT8-dependent fucosylation has been associated with impaired epithelial integrity and increased disease risk [95,98]. A more specific mechanistic link has been identified for FUT8, as loss of core fucosylation of the extracellular matrix protein SPARC impairs its collagen-binding capacity, thereby disrupting matrix homeostasis and contributing to COPD pathogenesis [98]. Impaired core fucosylation promotes emphysema through aberrant TGF-β/Smad signaling [99,100]. This is further supported by studies linking FUT8 deficiency or polymorphisms to emphysema susceptibility in both cigarette smoke-induced models and human genetic analyses [99,101]. Additionally, loss of FUT8-mediated core fucosylation disrupts TGF-β/Smad signaling and leads to abnormal lung development and emphysema-like changes [40]. In IPF, available genetic and experimental evidence suggests that distinct FUTs may exert divergent effects. Higher circulating FUT3 levels have been associated with reduced IPF risk, whereas FUT8-driven core fucosylation plays a central role in amplifying profibrotic signaling in IPF [43,60,102]. FUT8 enhances TGF-β receptor activation and downstream Smad signaling, promoting fibroblast-to-myofibroblast differentiation, extracellular matrix deposition, and tissue stiffening. Similar mechanisms have been implicated in pulmonary arterial hypertension (PAH), where FUT8-mediated fucosylation of growth factor receptors, including VEGFR, activates PI3K/AKT pathways, contributing to vascular remodeling and smooth muscle proliferation [103]. Collectively, these studies suggest that dysregulated fucosylation contributes to chronic lung disease progression by converging on epithelial integrity, metabolic reprogramming, extracellular matrix homeostasis, and vascular remodeling.

## 7. Fucosylation-Senescence Signaling in Lung Disease

**Figure 3.**
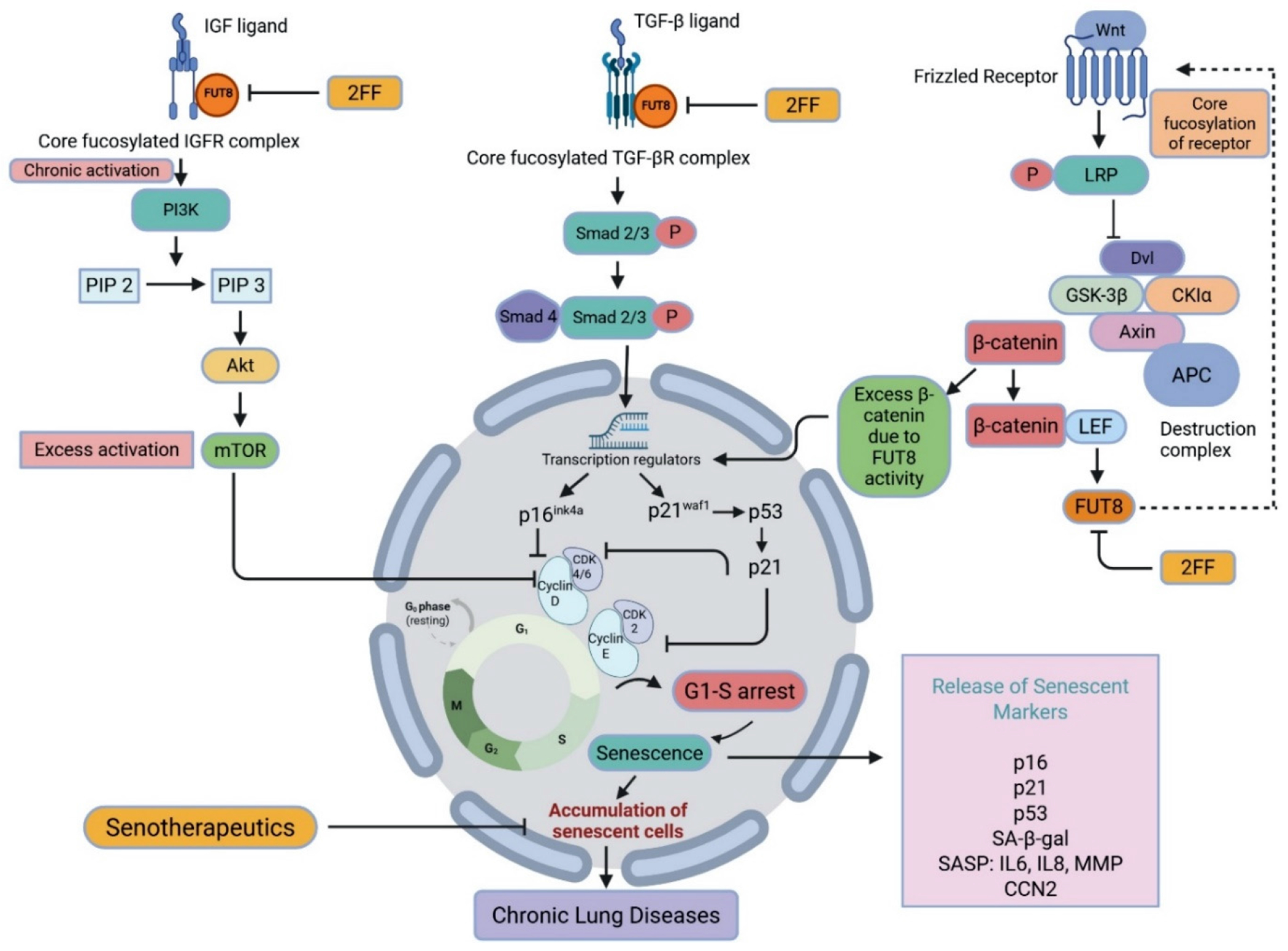
Core fucosylation regulates senescence-associated signaling in chronic lung diseases. FUT8-mediated core fucosylation enhances activation of key receptor pathways, including IGF-1R, TGF-βR, and Wnt co-receptors, thereby modulating cellular senescence. In the PI3K/Akt/mTOR pathway, fucosylation promotes sustained IGF-1R signaling and downstream Akt–mTOR activation. In the TGF-β/Smad pathway, it facilitates Smad2/3 phosphorylation and nuclear transcription of cell-cycle inhibitors (p16, p21, p53), leading to G1–S arrest. In the Wnt/β-catenin pathway, receptor fucosylation enhances β-catenin signaling and transcriptional activation. These pathways converge to drive cellular senescence, characterized by cell-cycle arrest, accumulation of senescent cells, and release of senescence-associated markers (e.g., SA-β-gal, IL-6, IL-8, MMPs). Pharmacological inhibition of fucosylation (e.g., 2FF) or senotherapeutics may attenuate these effects. Collectively, core fucosylation acts as an upstream regulator linking signaling activation to senescence and chronic lung disease progression.

Altered fucosylation has been repeatedly implicated in biological processes that converge on pathways central to senescence-associated pathology. In particular, fucosylation modifies the glycan structures of receptors, ligands, and adhesion molecules, thereby regulating their activity and influencing signaling pathways relevant to cellular senescence. Although direct evidence linking fucosylation to senescence in chronic lung diseases remains limited, emerging studies suggest potential intersections across several key signaling pathways, including TGF-β/Smad, PI3K/Akt/mTOR, and Wnt/β-catenin (Figure 3). These pathways are well established drivers of epithelial injury, chronic inflammation, fibroblast activation, and tissue remodeling in diseased lungs. Notably, many of their upstream receptors and signaling components are glycoproteins whose function may be modulated by fucosylation. Accordingly, dysregulated fucosylation may influence cellular senescence by altering receptor-dependent signaling output and downstream pathway activation. The following sections highlight representative signaling axes through which fucosylation may interface with senescence-associated programs in chronic lung diseases.

## 8. TGF-β/Smad Pathway

The TGF-β/Smad pathway is a central regulator of fibrosis, EMT, and senescence-associated tissue remodeling in chronic lung diseases [104–106]. Beyond its canonical role in fibrogenesis, accumulating evidence suggests that TGF-β signaling also functions as a key driver of cellular senescence programs in the injured lung microenvironment. Fucosylation, particularly FUT8-mediated core fucosylation, appears to be critical for efficient activation of the TGF-β receptor complex. Core fucosylation of N-glycans on TGF-β receptors enhances ligand-receptor affinity and stabilizes receptor conformation, thereby facilitating receptor dimerization and downstream signal propagation [99,100]. Consistent with this, FUT8 deficiency markedly impairs ligand binding and attenuates TGF-β signaling activity. FUT8-deficient mice exhibit abnormal lung development and an emphysema-like phenotype, accompanied by dysregulated TGF-β receptor activation [100]. These findings highlight a structural requirement for core fucosylation in maintaining TGF-β pathway integrity in the lung. Importantly, lung-specific studies further support a functional link between fucosylation and senescence. FUT8-mediated core fucosylation has been shown to promote alveolar epithelial cell senescence in pulmonary fibrosis through activation of TGF-β signaling, providing direct evidence that glycosylation-dependent modulation of receptor signaling can engage senescence-relevant profibrotic pathways [43].

Upon activation, TGF-β signaling induces phosphorylation of SMAD2 and SMAD3, followed by formation of SMAD2/3-SMAD4 transcriptional complexes that translocate to the nucleus. These complexes regulate a broad transcriptional program that extends beyond cell-cycle arrest to include induction of cyclin-dependent kinase inhibitors such as p21 and p16^INK4A^, suppression of proliferative genes, and activation of SASP components [105,107]. Through these coordinated outputs, TGF-β signaling reinforces stable growth arrest while simultaneously promoting a proinflammatory and profibrotic microenvironment. In addition, crosstalk between TGF-β/Smad signaling and other pathways, including PI3K/Akt/mTOR and Wnt/β-catenin, may further amplify senescence-associated phenotypes, suggesting that fucosylation-dependent modulation of TGF-β receptor activity could have broader network-level effects. Taken together, these findings support a model in which altered fucosylation intersects with cellular senescence through modulation of TGF-β/Smad signaling. By influencing receptor activation, signal strength, and downstream transcriptional programs, fucosylation may couple glycoprotein remodeling to growth arrest, SASP induction, and profibrotic tissue remodeling in chronic lung diseases.

## 9. PI3K/Akt/mTOR Pathway

The PI3K/Akt/mTOR signaling pathway plays a central role in chronic airway inflammation and tissue remodeling and has been extensively implicated in epithelial injury, dysregulated autophagy and apoptosis, and mitochondrial dysfunction in airway epithelial cells [108,109]. In parallel, accumulating evidence indicates that this pathway also contributes to the establishment and maintenance of senescence-associated programs in chronic lung diseases [110,111]. Persistent activation of mTOR has been linked to cellular senescence and COPD-like phenotypes, while enhanced Akt signaling has been associated with alveolar epithelial cell senescence in IPF [75,80]. Mechanistically, the PI3K/Akt/mTOR axis functions as a key integrator of growth factor signaling, nutrient sensing, and cellular stress responses, all of which are closely connected to senescence regulation. Sustained activation of this pathway promotes inhibition of autophagy, metabolic reprogramming, and increased protein synthesis, thereby reinforcing senescence-associated phenotypes and contributing to the development of a proinflammatory and profibrotic microenvironment [112]. Fucosylation may intersect with this pathway primarily through modulation of upstream receptor signaling. A particularly relevant example is the insulin-like growth factor-1 receptor (IGF-1R), a glycoprotein whose activity is sensitive to core fucosylation. Core fucosylation of IGF-1R enhances ligand binding and receptor activation, thereby amplifying downstream IGF-1/PI3K/Akt signaling [113,114]. In pulmonary fibrosis models, this modification has been shown to promote activation of the IGF-1/PI3K/Akt pathway and drive alveolar epithelial cell senescence. Importantly, Sun et al. demonstrated that inhibition of core fucosylation attenuates IGF-1-induced activation of PI3K/Akt signaling, reduces alveolar epithelial cell senescence, and alleviates pulmonary fibrosis, thereby providing direct evidence linking fucosylation to senescence through this signaling axis in the lung [60]. These findings support a model in which glycosylation-dependent regulation of receptor activity modulates downstream signaling intensity and duration, ultimately influencing senescence outcomes. In addition to IGF-1R, other receptor tyrosine kinases and cytokine receptors within the PI3K/Akt/mTOR network are also glycosylated, raising the possibility that fucosylation exerts broader regulatory effects on this pathway. Furthermore, age-related increases in FUT8 expression and activity have been reported in multiple tissues, including the liver, suggesting that enhanced core fucosylation may represent a conserved mechanism contributing to age-associated activation of PI3K/Akt signaling across organ systems [115]. Collectively, these observations indicate that the PI3K/Akt/mTOR axis represents a critical signaling hub through which core fucosylation may influence cellular senescence in chronic lung disease. By modulating receptor activation, signal amplification, and downstream metabolic and autophagic programs, fucosylation may couple glycan remodeling to senescence-associated epithelial dysfunction and fibrotic remodeling.

## 10. Wnt/β-Catenin Pathway

Accumulating evidence indicates that Wnt/β-catenin signaling plays a critical role in epithelial cell fate determination, regeneration, and tissue remodeling in chronic lung diseases. In addition to its well-established function in developmental and repair processes, dysregulated or sustained activation of Wnt/β-catenin signaling has been increasingly linked to cellular senescence and aberrant epithelial reprogramming in the diseased lung. In lung epithelial cells, persistent Wnt/β-catenin activity has been associated with senescence phenotypes and maladaptive remodeling, supporting a role for this pathway in senescence-associated lung pathology [73]. Mechanistically, activation of the Wnt/β-catenin pathway leads to stabilization and nuclear translocation of β-catenin, where it interacts with TCF/LEF transcription factors to regulate gene expression programs involved in proliferation, stemness, and differentiation [116]. Under conditions of chronic activation, this signaling axis may shift from regenerative to maladaptive outputs, promoting epithelial dysfunction, altered cell fate, and senescence-associated phenotypes.

Emerging evidence suggests that protein fucosylation may modulate key components of the Wnt/β-catenin pathway at the receptor level. For instance, FUT8 deficiency in mouse embryonic fibroblasts results in enhanced Wnt/β-catenin signaling and upregulation of Wnt target genes, indicating that core fucosylation may normally act to constrain pathway activation [117]. In addition, cell-surface fucosylation of the Wnt co-receptor LRP6 has been shown to regulate Wnt ligand–receptor interactions and downstream signaling activity, providing direct evidence that receptor glycosylation can influence pathway output [118]. These findings support a model in which fucosylation modulates Wnt signaling by structurally and functionally regulating receptor complexes. Although direct evidence linking FUT-dependent fucosylation to Wnt-driven senescence in chronic lung disease remains limited, the convergence of these observations suggests a plausible mechanistic intersection. By regulating receptor activation and signaling amplitude, altered fucosylation may influence β-catenin–dependent transcriptional programs that govern epithelial plasticity, senescence, and tissue remodeling. Taken together, the Wnt/β-catenin pathway represents an additional signaling axis through which fucosylation may interface with cellular senescence.

## 11. Emerging Therapeutic Opportunities in Chronic Lung Diseases

Protein glycosylation remodeling is a common feature of multiple pathological states, raising the possibility that fucosylation-related alterations may serve as clinically informative biomarkers in chronic lung diseases. In this context, profiling fucosyltransferase expression and protein-specific fucosylation patterns may enable disease stratification, prognostic assessment, and monitoring of pathological remodeling. Supporting this concept, increased fucosylation of serum surfactant protein D has been associated with COPD development and emphysema severity, whereas genetically elevated circulating FUT3 levels have been linked to reduced disease risk in IPF [119,120]. Beyond biomarker development, accumulating evidence suggests that altered fucosylation is not merely a downstream feature of disease but may act as an upstream regulator of senescence-associated signaling networks. Through modulation of receptor-dependent pathways such as TGF-β/Smad, PI3K/Akt/mTOR, and Wnt/β-catenin, dysregulated fucosylation may reinforce cellular senescence and amplify its downstream pathological consequences, including chronic inflammation, epithelial dysfunction, and fibrotic remodeling. This interaction is likely to be particularly relevant in chronic inflammatory and fibrotic lung diseases, such as COPD, severe asthma, and IPF, especially in aging populations in whom senescence-associated processes are more pronounced.

These observations suggest that the fucosylation–senescence axis may represent a tractable therapeutic target. If fucosylation is confirmed to function as a disease-relevant driver of senescence-associated signaling, targeting fucosyltransferases, particularly FUT8-mediated core fucosylation, could attenuate upstream pathway activation and limit the establishment of pro-senescent cellular states. In parallel, senescence-directed interventions, including senolytic approaches to eliminate established senescent cells and senomorphic strategies to suppress SASP activity, may provide complementary means to mitigate downstream inflammatory and profibrotic responses.

## 12. Conclusions and Future Perspectives

Accumulating evidence indicates that α(1,2)-fucosylation is an important regulator of cellular signaling, immune responses, tissue remodeling, and senescence-related processes in chronic lung diseases. Although the mechanistic links between fucosylation and senescence remain incompletely understood, emerging studies suggest that aberrant fucosylation may influence key pathways involved in chronic inflammation, epithelial dysfunction, fibrosis, and age-associated tissue remodeling. These findings support a translational framework in which glycosylation-based biomarkers, fucosylation-targeted interventions, and senescencemodulating therapies are integrated to address distinct but interconnected levels of disease pathogenesis. Such a multi-layered approach may offer advantages over single-pathway targeting by simultaneously modulating upstream signaling networks and downstream cellular outcomes. Furthermore, while the concept of distinct glycan signatures underlying beneficial and maladaptive senescence is highly intriguing, the available literature remains limited. Future studies are therefore needed to define the glycan landscapes associated with different senescent states and to determine their functional significance in health and disease by integrating single-cell transcriptomics, glycoproteomics, spatial profiling, and other multi-omics approaches. These investigations may help identify cell type-specific fucosylation signatures associated with senescence and provide mechanistic insights into their contribution to the initiation and progression of chronic lung diseases. Future work should also establish the specific cell types, disease stages, and signaling contexts in which the fucosylation–senescence axis is most biologically and therapeutically relevant.

In addition, age-related alterations in glycosylation are increasingly recognized as contributors to cellular dysfunction and senescence-associated signaling pathways. Altered fucosylation patterns have been linked to chronic low-grade inflammation (“inflammaging”) and a variety of age-associated disorders, including cardiovascular disease, ischemic stroke, and neurodegenerative diseases [121,122]. Moreover, age-dependent glycosylation signatures are being incorporated into emerging “glycan clocks” as indicators of biological age and predictors of age-related functional decline [123,124]. Together, these findings suggest that dysregulated fucosylation may represent an important molecular link connecting aging, cellular senescence, and chronic inflammatory diseases, including chronic lung disorders.

As technologies for glycan profiling and functional glycomics continue to advance, a more comprehensive understanding of the glycosylation landscape of senescent cells may reveal novel biomarkers and therapeutic opportunities. Ultimately, elucidating how fucosylation shapes senescence programs across different cell populations and disease contexts may facilitate the development of precision glyco-senolytic strategies aimed at translating insights from glycosylation biology into clinically effective strategies for the prevention and treatment of chronic lung diseases.

## Figures and Tables

**Table 1.** Key signaling pathways driving cellular senescence in airway cells in chronic lung diseases.

Key Signaling Pathways	Chronic Lung Diseases	Affected Airway Cell Types	Role of Signaling Pathway
TGF-β/Smad	IPF	Bronchial epithelial cells	TGF-β/Smad signaling induces epithelial cell senescence, which impairs epithelial repair and promotes profibrotic remodeling in IPF [65].
		Alveolar epithelial cells type II (ATII)	p53 activation during AT2 cell senescence promotes an autocrine TGF-β-positive feedback loop, sustaining TGF-β/Smad signaling and driving myofibroblast differentiation in IPF [66].
			TGF-β1 induces senescence and profibrotic transition in AT2 cells and lung fibroblasts [67].
		Lung fibroblasts	TGF-β stimulation induces senescence in IPF lung fibroblasts, increasing collagen I and promoting fibroblast-myofibroblast transition. [68].
			TGF-β1 promotes lung fibroblast proliferation and senescence in IPF [67].
	COPD/Emphysema	Small airway fibroblasts	Small airway fibroblasts in COPD patients demonstrated senescence and upregulated TGF-β signaling [69].
		Lung fibroblasts	FOXO1 promotes cigarette smoke condensate-induced lung fibroblast senescence and fibrosis by activating the TGF-β1/Smad2/3 signaling pathway [70].
			Reduced LTBP4 expression is implicated in age-related emphysema pathogenesis through dysregulated latent TGF-β signaling and impaired extracellular matrix homeostasis [71].
	Pulmonary hypertension	Pulmonary artery smooth muscle cells (PASMC)	Senescent PASMCs promote pulmonary hypertension through paracrine secretion of remodeling-associated mediators, including TGF-β [72].
Wnt/β- catenin IPF	Asthma	Alveolar epithelial cells	Wnt/β-catenin signaling mediates senescent epithelial cell-induced fibroblast activation by promoting β-catenin-dependent Nanog expression [73].
		Alveolar epithelial type II cells	Chronic WNT/β-catenin signaling induces cellular senescence in ATII cells, and promotes profibrotic epithelial reprogramming [73].
		Bronchial fibroblasts	Deranged Wnt/β-catenin signaling is associated with abnormal senescence-related stress responses and dysregulated repair programs in asthmatic bronchial epithelium and fibroblasts [74].
	COPD	Small airway fibroblasts	Small airway fibroblasts in COPD patients demonstrated senescence and upregulated Wnt/β-catenin signaling [69].
PI3K/AKT/mTOR	IPF	Alveolar epithelial cells	PTEN loss promotes alveolar epithelial cell senescence in pulmonary fibrosis through Akt pathway activation [75].
		Alveolar epithelial cells	Core fucosylation promotes alveolar epithelial cell senescence and pulmonary fibrosis by enhancing IGF1/PI3K/AKT signaling [15].
	Asthma	Airway epithelial cells	mTOR signaling mediates cigarette smoke-induced cellular senescence and thereby promotes asthma development [76].
	COPD/Emphysema	Bronchial epithelial cells	PDK1 inhibition reduces airway epithelial cell autophagy and senescence through suppression of the PI3K/AKT pathway in cigarette smoke-induced emphysema [77].
			Erythromycin attenuates oxidative stress-induced cellular senescence via the PI3K/AKT/mTOR signalling pathway in COPD [78].
		Alveolar epithelial type II cells	p16 senescence pathway promotes AECII proliferation and regeneration by upregulating IGF1/Akt signalling pathway in emphysema [79].
		Pulmonary endothelial cells Alveolar epithelial cells	mTOR pathway activation drives senescence in lung vascular and alveolar epithelial cells, thereby promoting emphysema, pulmonary hypertension, and inflammation [80,81].
	Pulmonary hypertension	Pulmonary artery smooth muscle cells (PASMC)	mTOR signalling promotes PASMC senescence and pulmonary vascular remodelling in pulmonary hypertension [82].
		Pulmonary vascular endothelial cells	Activation of PI3K/AKT/mTOR signalling mediates miR-21-induced endothelial senescence and dysfunction in pulmonary hypertension [83].
MAPK p38	IPF	Alveolar epithelial cells	Persistent DNA damage activates p38 MAPK-associated responses and promotes SASP development in alveolar epithelial cells after bleomycin-induced lung injury [84].
		Lung fibroblasts	p38 MAPK promotes replicative senescence-associated profibrotic gene expression in lung fibroblasts [84].
		Airway epithelial cells	Airway epithelial cell senescence impairs repair and promotes p38 MAPK-dependent inflammation after airway injury [85].
	COPD	Bronchial epithelial cells	FOXA2 protects against cigarette smoke-induced cellular senescence and inflammation through suppression of p38 and Erk1/2 MAPK signaling [86].
JAK/STAT	IPF	Alveolar type II epithelial cells Lung fibroblasts	Activated JAK2/STAT3 signalling contributes to idiopathic pulmonary fibrosis by promoting profibrotic and senescence-associated responses [87].
		Lung fibroblasts	Dysregulated STAT3 activation induces fibroblast senescence and progression of IPF [88].
	COPD	Bronchial epithelial cells	Interferon drives chronic airway inflammation and bronchial epithelium senescence via JAK/STAT pathway in COPD [89].
	Asthma	Bronchial epithelial cells	TSLP promotes airway remodelling in asthma by inducing cellular senescence via STAT3 signaling [90].
NF-κB	IPF	Alveolar epithelial cells	PTEN loss induces alveolar epithelial cell senescence in pulmonary fibrosis through NF-κ activation [91].

IPF: Idiopathic pulmonary fibrosis, COPD: Chronic obstructive pulmonary disease, ATII: Alveolar epithelial cells type II, PASMC: Pulmonary artery smooth muscle cell.

## Data Availability

No datasets were generated or analyzed during the current study.
